# Ventriculoperitoneal shunt after previous endoscopic third ventriculostomy: does ETV improve shunt survival?

**DOI:** 10.1186/1743-8454-7-S1-S14

**Published:** 2010-12-15

**Authors:** Ash Singhal, Tia Liu, Doug Cochrane, Paul Steinbok

**Affiliations:** 1 Department of Pediatric Surgery, British Columbia Children's Hospital, University of British Columbia, 4480 Oak Street, Room K3-159, Vancouver, British Columbia, V6H 3V4, Canada

## Background

Endoscopic Third Ventriculostomy (ETV) has been used as an alternative to Ventriculoperitoneal Shunt (VPS), but some patients “fail” ETV and subsequently require VPS insertion. However, little is known about the subsequent need for shunt revision in these patients. The current study aims to determine if there is a difference in shunt failure rates in patients who have had previous ETV, as compared with patients who have never had previous ETV.

## Materials and methods

A case-control study was performed. We identified all patients treated with ETV at our institution who subsequently required VPS. Control subjects were selected, matched for age and hydrocephalus etiology. A survival analysis was performed for the VPS, to determine if there was any difference in shunt survival in ETV patients vs. non-ETV patients.

## Results

We identified 17 patients with “failed” ETV who went on to require VPS, and selected 34 control subjects. Age and hydrocephalus etiology were similar in the case and control groups. There were 8 deaths (3 in the ETV group and 5 the non-ETV group), generally in brain tumor patients, and these cases were excluded. Of 14 ETV patients, 71% (10 patients) never required a subsequent revision (mean follow-up 5.9 years), and 29% (4 patients) required revisions (mean time to first revision was 1.5 years). In 29 control subjects, 34% (10 patients) never required subsequent revision (mean follow-up 8.9 years), and 66% required revisions (mean time to first revision was 1.5 years). The shunt after ETV was significantly more likely to survive (p=0.023) than the shunt in the non-ETV group.

## Conclusions

VPS in patients with previous “failed” ETV appear to have better survival than VPS in patients who have never had ETV. This has interesting implications in considering the potential benefit of ETV, even when a VPS is subsequently necessary.

